# Synthesis of Air-Stable Cu Nanoparticles Using Laser Reduction in Liquid

**DOI:** 10.3390/nano11030814

**Published:** 2021-03-23

**Authors:** Ashish Nag, Laysa Mariela Frias Batista, Katharine Moore Tibbetts

**Affiliations:** Department of Chemistry, Virginia Commonwealth University, Richmond, VA 23284, USA; nagab@vcu.edu (A.N.); friasbatistlm@vcu.edu (L.M.F.B.)

**Keywords:** laser synthesis, laser reduction in liquid, copper nanoparticles, para-nitrophenol

## Abstract

We report the synthesis of air-stable Cu nanoparticles (NPs) using the bottom-up laser reduction in liquid method. Precursor solutions of copper acetlyacetonate in a mixture of methanol and isopropyl alcohol were irradiated with femtosecond laser pulses to produce Cu NPs. The Cu NPs were left at ambient conditions and analyzed at different ages up to seven days. TEM analysis indicates a broad size distribution of spherical NPs surrounded by a carbon matrix, with the majority of the NPs less than 10 nm and small numbers of large particles up to ∼100 nm in diameter. XRD collected over seven days confirmed the presence of fcc-Cu NPs, with some amorphous Cu_2_O, indicating the stability of the zero-valent Cu phase. Raman, FTIR, and XPS data for oxygen and carbon regions put together indicated the presence of a graphite oxide-like carbon matrix with oxygen functional groups that developed within the first 24 h after synthesis. The Cu NPs were highly active towards the model catalytic reaction of *para*-nitrophenol reduction in the presence of NaBH_4_.

## 1. Introduction

Laser synthesis techniques have emerged over the last decade as reliable methods for producing pure nanomaterials (NMs) [1,2,3]. Laser synthesis has several advantages over traditional chemical synthesis methods including: facile generation of metastable phases and bonding environments, rapid conversion of precursors to NM products, and avoidance of capping ligands. For instance, alloys of immiscible metals can be formed [4], reactions can be completed in seconds [5], and grams per hour synthesis yields can be attained with high-repetition rate lasers [6]. Moreover, laser synthesis techniques are considered ’green’ because they do not employ toxic chemical reducing agents or surfactants and produce little chemical waste [7].

The lack of otherwise required capping ligands results in high purity nanoparticles that are important to several applications. Biofunctional assemblies can be produced by functionalizing laser-generated nanoparticles (NPs) for in-vitro applications [8]. The absence of ligands makes more active sites available for catalysis, resulting in laser-synthesized NPs often having higher activities than their conventional counterparts [9,10,11]. Higher purity also makes laser-synthesized NPs attractive candidates for other biomedical applications and as references for modeling chemical reactions [12,13,14,15]. In addition, photoluminescence can be introduced to the NPs by in-situ generation of carbon shells through laser-induced decomposition of organic solvents [16,17].

Cu NPs in particular are of high interest both due to the natural abundance of copper and their myriad applications in catalysis, electronics, and biology [18,19,20,21,22]. However, conventional wet-chemical methods used to synthesize Cu-based NPs require toxic solvents, reducing agents, or both [23,24,25]. Hence, greener synthesis routes to Cu NPs are of primary importance. To address this need, pulsed laser ablation in liquid (PLAL) has been widely employed to generate Cu NPs [26,27,28,29,30]. In these syntheses, copper oxides are a major product when ablation is conducted in water [26,27,28,29], and Cu${}^{0}$ phases are only stable when the ablation liquid contains an organic solvent, such as acetone, methanol, or ethanol [28,29,30].

The *top-down* PLAL method is by far the most common laser synthesis technique used to produce colloidal metal NPs [1]. PLAL involves focusing of laser beam on the surface of a solid or powdered target immersed in a solvent, causing the removal of target material from the surface and its coalescence into colloidal NPs. However, NP products from PLAL in many cases exhibit bimodal size distributions due to the ejection of both small clusters and large droplets from the surface [31,32,33]. An alternative method is *bottom-up* laser reduction in liquid (LRL), which involves focusing picosecond (ps, 10${}^{-12}$ s) or femtosecond (fs, 10${}^{-15}$ s) laser pulses into a solution of molecular precursors to generate a dense plasma containing electrons that reduce metal ions to colloidal NPs. LRL can enable superior control over Au NP sizes in a single step when the chemistry of the precursor solution and laser irradiation conditions are carefully controlled [34,35].

A current limitation to the wide use of LRL is that the vast majority of studies reported to date focus on the easily reduced noble metals Au [34,35,36,37,38], Ag [39,40,41], Pt [42], and their alloys [43,44,45] because these metals are resistant to oxidation. Unlike in PLAL where the zero-valent metal is present in the initial target, metal NP formation in LRL requires chemical reactions between the molecular precursor(s) and the reactive species in LRL plasma. In aqueous solution, the major species are hydrated electrons (e_aq_−) and hydroxyl radicals (OH·) [38]. Whereas e_aq_− are exceptionally strong reducing agents towards metal ions, OH· radicals and their recombination product H_2_O_2_ can back-oxidize zero-valent metal atoms. Although Au is resistant to back-oxidation and HAuCl_4_ precursor can be reduced by H_2_O_2_ [37], effective production of Ag-containing NPs by LRL requires the addition of OH· scavengers, such as ammonia or isopropyl alcohol, to prevent back-oxidation of Ag [40,41,45]. Moreover, the few LRL studies on the non-noble metal Fe report production of only Fe oxides [46,47].

The detrimental effects of reactive oxygen species on Cu NP synthesis have been widely known since Dhas et al. [48] reported that OH· and H_2_O_2_ formed during sonochemical synthesis lead to the formation of copper oxides. In PLAL of Cu metal in water, Cu oxides are formed due to the presence of OH· radicals in the laser plasma from water, dissolved O_2_, or both [28]. Oxide formation during PLAL can be hindered through the use of organic solvents [27,28,29,30], which also can result in the formation of a protective carbon shell around the Cu NPs [27,28]. On the basis of these results, we designed an air-free LRL synthesis route to Cu NPs using an isopropyl alcohol/methanol solvent mixture. We report, to the best of our knowledge, the first LRL synthesis of air-stable Cu NPs and their catalytic activity towards reduction of *para*-nitrophenol in the presence of NaBH_4_.

## 2. Materials and Methods

### 2.1. Materials

Copper acetylacetonate (Cu(acac)_2_, Acros Organics, Fair Lawn, NJ, USA), isopropyl alcohol (IPA, Fisher Scientific, Waltham, MA, USA), methanol (MeOH, Fisher Scientific, Waltham, MA, USA), sodium borohydride (NaBH_4_, Acros Organics, Fair Lawn, NJ, USA), and *para*-nitrophenol (PNP, Acros Organics, Fair Lawn, NJ, USA) were used as received.

### 2.2. Cu NP synthesis

A working solution of 0.8 mM Cu(acac)_2_ in 25%/75% (*v/v*) MeOH/IPA solvent was purged with nitrogen for 20 min before 3.0 mL of the solution was transferred to a nitrogen-filled 10 × 10 × 40 mm quartz fluorimeter cuvette. The solution was then irradiated with laser pulses to yield Cu NPs. Upon formation, Cu NPs were exposed to air and analyzed at different time intervals (0 h, 3 h, 24 h, and 7 days) to study the aging of NPs. By 24 h after synthesis, all the Cu NPs are precipitated out of the solution but can be redispersed via brief sonication.

### 2.3. Instrumentation

#### 2.3.1. Laser Synthesis

The experimental setup is described elsewhere [38]. Briefly, samples were irradiated using a commercial titanium-sapphire chirped-pulse amplifier (Astrella, Coherent, Inc., Santa Clara, CA, USA), delivering 30 fs pulses, with the bandwidth centered at 800 nm and a repetition rate of 1 kHz; 2 mJ laser pulses were focused using a f=5 cm aspheric lens into the center of the sample cuvette to produce a peak irradiance of 5 × $10^{16}$ Wcm${}^{-2}$. Details for calculating peak irradiance can be found in Ref. [38]. The sealed cuvettes containing Cu(acac)_2_ solution were irradiated for 10 min.

#### 2.3.2. UV-Vis Spectroscopy

Conversion of precursors to Cu NPs was monitored using a home-built in-situ UV-vis spectrophotometer described in Ref. [38] and the irradiation was stopped when no further growth in Cu surface plasmon resonance (SPR) was observed. The catalytic performance of Cu NPs was tested for the reduction of PNP with NaBH_4_ (see Section 2.4) by employing a second home-built in-situ UV-vis spectrophotometer described in Ref. [49]. Finally, to observe the effect of aging of Cu NPs, absorbance data was recorded using Agilent 8453 UV-vis spectrophotometer at times up to 7 days after synthesis.

#### 2.3.3. Transmission Electron Microscopy (TEM)

Cu NPs were visualized using TEM (JEOL JEM-1230 TEM) at 120 kV. A diluted solution of colloidal Cu NPs was drop-casted onto carbon-coated grids (Structure Probe, Inc., West Chester, PA, USA) and left to dry for 24 h or longer. Average sizes and size distributions were measured using ImageJ software. At least 350 particles from images of three separate areas of a TEM grid were evaluated.

#### 2.3.4. X-ray Photoelectron Spectroscopy (XPS)

XP spectra were collected on a PHI VersaProbe III Scanning XPS Microprobe with a monochromated Al Kα X-ray source (1486.6 eV), with a typical resolution of 0.4–0.5 eV. Survey scans and high resolution scans were collected with pass energies of 280 eV and 26 eV, respectively. Charge neutralization was done by running an ion gun and a flood gun during sample analysis. The measurement spot diameter was 200 µm with take off angle of $90^{\circ }$ and the detector at $45^{\circ }$. Spectral analysis was carried out using PHI Multipak XPS software with 70% Gaussian/Lorentzian convolution to fit each spectral peak. Samples were prepared by drop-casting Cu NPs on a gold-sputtered silicon wafer, followed by drying under vacuum for at least 24 h. All spectra were corrected using a Au4f peak shift to center at 84.0 eV.

#### 2.3.5. FTIR Spectroscopy

A Thermo Scientific Nicolet iS50 FTIR spectrometer equipped with a mid- and far-IR-capable diamond ATR was used to record FTIR spectra. All spectra were obtained using 32 scans in the range from 4000 to 400 cm${}^{-1}$ with 4 cm${}^{-1}$ resolution. Cu NPs were directly drop-casted on to the diamond crystal.

#### 2.3.6. X-ray Diffraction (XRD)

XRD data was collected on a Panalytical Empyrean Diffractometer with CuKα radiation (0.15418 nm) at 40 kV and 45 mA, with scanning angle (2θ) of 30–80${}^{\circ }$ and a gonio focusing geometry. Sample preparation involved drop-casting of Cu NPs on a low-background silicon substrate, followed by drying under vacuum for at least 24 h.

#### 2.3.7. Raman Spectroscopy

Raman spectra were recorded on a Thermo Scientific DXR3 SmartRaman Spectrometer involving a 532 nm excitation laser. All spectra obtained were averaged over 32 scans in the range of 50 to 3500 cm${}^{-1}$ with 5 cm${}^{-1}$ resolution. Samples were prepared by drop-casting Cu NPs on a silicon wafer, followed by drying under vacuum for at least 24 h.

### 2.4. Catalytic Reduction of Para-Nitrophenol (PNP)

PNP reduction reactions were carried out in a home-built in situ UV-vis spectrometer setup described elsewhere [35] at different time intervals after laser synthesis (0 h, 3 h, 24 h, and 7 days). Spectra were recorded every 2.4 s using LabVIEW software (National Instruments). A solution containing a final concentration of 0.1 mM PNP and 10 mM of freshly prepared NaBH_4_ was prepared in a 10 × 10 × 40 mm quartz fluorimeter cuvette with a magnetic stir bar, resulting in the formation of *p*-nitrophenolate ion with UV-vis absorbance at 400 nm. Prior to the addition of the catalyst, this peak was observed for 24 s to confirm no reaction occurred in the absence of a catalyst. After this period, 300 µL of the Cu NP catalyst was added, triggering the reduction of PNP. Data collection was terminated when the absorbance at 400 nm (*p*-nitrophenolate ion) had disappeared.

## 3. Results

### 3.1. Physical Characterization

**Figure 1:**
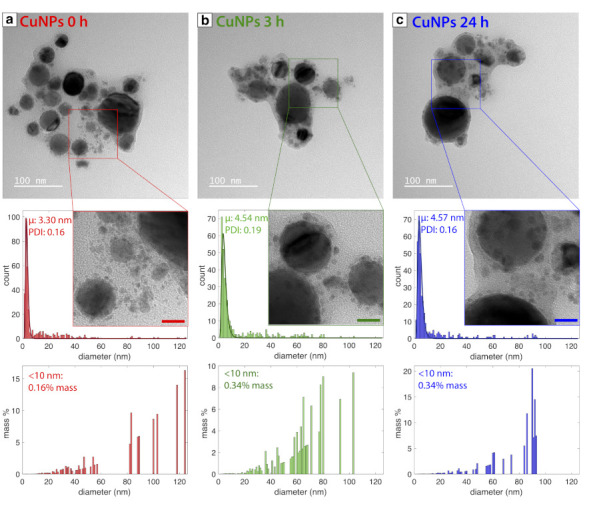
TEM images and corresponding number-weighted and mass-weighted size distributions for samples analyzed (**a**) 0 h, (**b**) 3 h, and (**c**) 24 h after synthesis. Scale bars in magnified regions are 20 nm.

Figure 1 shows TEM images and size distributions for Cu NPs samples obtained 0 h (a), 3 h (b), and 24 h (c) after laser synthesis. In all images, small <10 nm NPs surrounding significantly larger NPs are observed. Fitting the size distributions to a log-normal function primarily captured the small NPs, producing mean diameters of 3.30 nm (PDI = 0.16), 4.54 nm (PDI = 0.19), and 4.57 nm (PDI = 0.16) for 0 h, 3 h, and 24 h samples, respectively. Despite the large numbers of small NPs, they make up a tiny fraction of the overall mass of Cu NPs, as evident in the mass-weighted size distributions. Nevertheless, the contribution of NPs smaller than 10 nm to the overall mass slightly increased from 0.16 % at 0 h to 0.34 % at 3 h and 24 h. The slight size increase in the small NPs after the initial synthesis was also observed for Fe oxide NPs synthesized by LRL from Fe(acac)_3_ in hexane [47], although, in that work, no large NPs were observed. A carbon shell around clusters of Cu NPs is also visible in Figure 1, particularly for the 3 h and 24 h samples. Similar carbon shells have been observed for Cu NPs produced by PLAL in acetone [28] and methanol [27].

**Figure 2:**
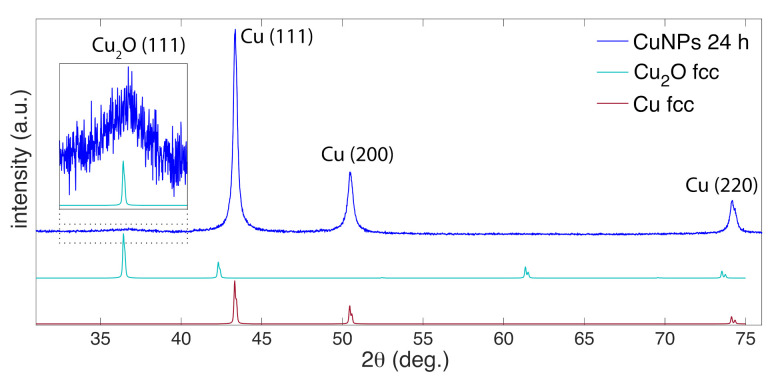
XRD spectrum of 24 h sample (blue), with references for Cu_2_O (cyan) and Cu (dark red). Inset magnifies a broad peak observed in the spectra for 24 h sample indexed to Cu_2_O (111).

Figure 2 shows the XRD spectrum of the 24 h sample. Intense diffraction peaks observed at 43.35${}^{\circ }$, 50.47${}^{\circ }$, and 74.18${}^{\circ }$ were indexed to (111), (200), and (220) planes of fcc-Cu (JCPDS no. 01-085-1326), respectively. A broad feature centered at 36.85${}^{\circ }$ indexed to cubic-Cu_2_O(111) (JCPDS no. 00-005-0667) was also observed. Using the Scherrer equation, the crystallite sizes were calculated to be 29.89 nm for the Cu(111) [50]. Thus, we can infer that the large NPs observed in Figure 1 are Cu metal NPs, whereas Cu_2_O exists as an amorphous thin layer around the Cu NPs, as the smaller NPs, or both.

**Figure 3:**
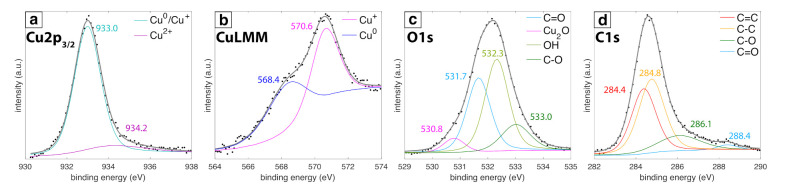
XPS spectra of (**a**) Cu2p${}_{3/2}$, (**b**) CuLMM, (**c**) O1s and (**d**) C1s regions with fitted peaks for 24 h samples.

Figure 3 shows XP spectra obtained for the 24 h sample. Two peaks in the Cu2p${}_{3/2}$ region (Figure 3a) at 933.0 eV (cyan) and 934.2 eV (violet) were assigned to either Cu${}^{0}$ or Cu${}^{+}$ and Cu${}^{2+}$, respectively [51]. To distinguish between Cu${}^{0}$ and Cu${}^{+}$, the CuLMM region was analyzed (Figure 3b) and resulted in two peaks at 568.4 eV (blue) and 570.6 eV (magenta) assigned to Cu${}^{0}$ and Cu${}^{+}$, respectively [51]. The presence of both Cu${}^{0}$ and Cu${}^{+}$ in the XP spectra is consistent with the XRD results (Figure 2). In the O1s region (Figure 3c), the peaks at 530.8 eV (magenta), 531.7 eV (light blue), 532.2 eV (light green), and 533.0 eV (dark green) were assigned to Cu–O, C=O, –OH and C–O species, respectively [52,53]. In the C1s region (Figure 3d), peaks at 284.4 eV (red), 284.8 eV (orange), 286.1 eV (dark green), and 288.4 eV (light blue) are assigned to C=C, C–C, C–O, and C=O species, respectively [53]. This collection of carbon species is associated with the carbon shell observed around the Cu NPs (Figure 1).

**Figure 4:**
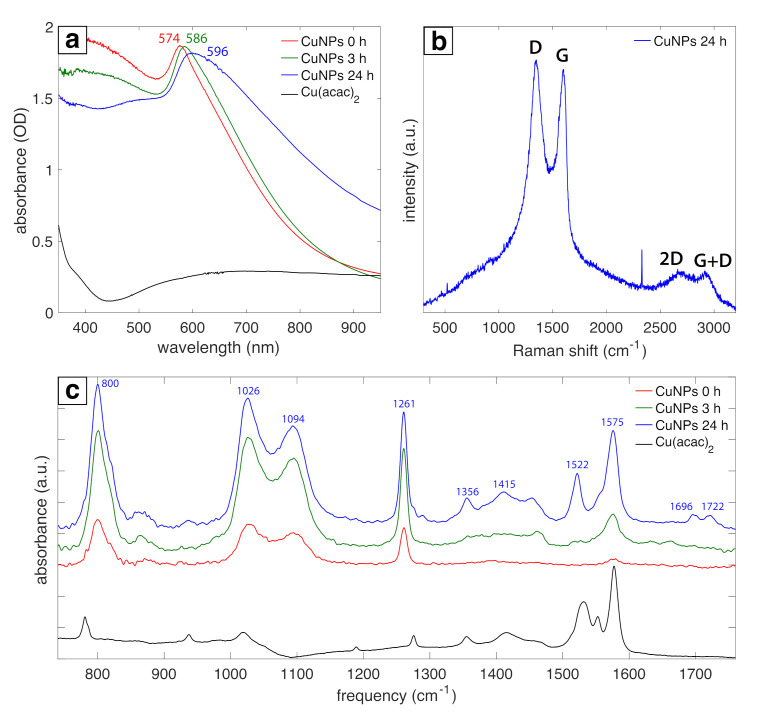
(**a**) UV-vis absorbance spectra for 0 h, 3 h, 24 h samples and precursor. (**b**) Raman spectra for 24 h samples. (**c**) FTIR spectra for 0 h, 3 h, 24 h samples and precursor.

Spectral characterization of the Cu NPs (Figure 4) confirms the chemical species assigned in Figure 2 and Figure 3. Figure 4a shows the UV-vis absorbance data collected at 0 h, 3 h, and 24 h after synthesis. An absorbance peak at 574 nm assigned to the Cu surface plasmon resonance (SPR) was observed at 0 h, followed by broadening and red-shifting to 586 nm and 596 nm at 3 h and 24 h, respectively. These changes in the absorbance peaks are associated with the oxidation of Cu to Cu_2_O over time [54] and likely indicate the formation of a Cu_2_O shell around the large Cu NPs on the basis of the XRD spectrum (Figure 2). Raman spectra shown in Figure 4b closely resemble graphite oxide spectra [55]. The intense G band at 1600 cm${}^{-1}$ and D band at 1350 cm${}^{-1}$ correspond to the in-plane vibrational modes of sp${}^{2}$-hybridized carbon atoms and structural disorder due to functionalization, respectively [56]. Moreover, the weak and broad 2D and D + G bands at 2680 cm${}^{-1}$ and 2910 cm${}^{-1}$ arise from disorder due to formation of oxygen functional groups [55]. The presence of oxygen functional groups is confirmed by the FTIR spectra (Figure 4c) with peaks indicating C=O stretch of COOH groups at 1722 and 1696 cm${}^{-1}$, COO${}^{-}$ stretch at 1415, 1522, 1575 cm${}^{-1}$, O–H deformations of C–OH groups at 1356 cm${}^{-1}$, C–O stretch of epoxide groups at 1261 cm${}^{-1}$, C–O stretch at 1000–1100 cm${}^{-1}$, and C–H bend at 800 cm${}^{-1}$ [56,57], although some of these bands may be overlapped with vibrational modes of other functional groups. Notably, these IR peaks grow in intensity over the course of 24 h after synthesis, suggesting that the carbon shell formation occurs mostly after laser irradiation is terminated. Overall, these data and the XP spectra for C1s and O1s (Figure 3a,b) indicate that the carbon shell around the Cu NPs consists of disordered graphite oxide-like structures that contain multiple different functional groups.

### 3.2. Catalytic Activity of Cu NPs

**Figure 5:**
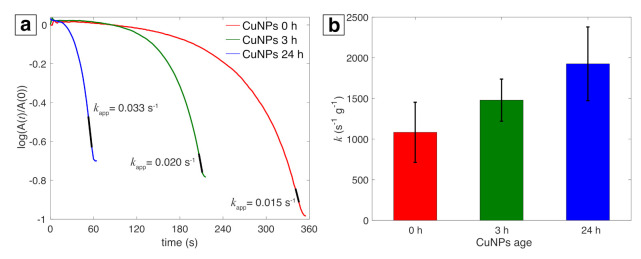
(**a**) Time dependence of the absorbance of *p*-nitrophenolate ions at 400 nm for a representative sample at 0 h, 3 h, and 24 h; black portion of line is where k${}_{app}$ (s${}^{-1}$) is extracted. (**b**) Specific rate constants (s${}^{-1}$g${}^{-1}$) with error bars generated over three runs for 0 h, 3 h, 24 h samples each.

Reduction of PNP by NaBH_4_ is a commonly used model reaction to test catalytic activity of metal NPs by employing UV-vis spectroscopy to monitor the decrease in absorbance of the *p*-nitrophenolate ion at 400 nm, which allows for convenient determination of pseudo-first-order rate constants when excess NaBH_4_ is present [58,59]. Figure 5a shows representative kinetic curves obtained from the ratio of the natural log of the 400 nm absorbance feature at time *t*, A(t), to the initial absorbance, A(0), as a function of reaction time *t* for the 0 h, 3 h, and 24 h samples. The slopes obtained from the linear regions of the curves (shown in black) give the apparent rate constants $\left(k_{\mathrm{app}}\right)$. The values of $k_{\mathrm{app}}$ were averaged over three different samples for all three post-synthesis times and then converted to the mass-specific rate constants (*k*) of 1084, 1479, and 1927 s${}^{-1}$g${}^{-1}$ for 0 h, 3 h, and 24 h samples (Figure 5b). The specific rate at 3 h is comparable to the rate of 1490 s${}^{-1}$g${}^{-1}$ for Cu NPs synthesized by PLAL in a water-ethanol mixture [30]. Although the rate constant is higher at 24 h, this increase could be due to some evaporation of the solvent over time, resulting in concentration of the Cu NPs and an artificially high measured rate.

### 3.3. Stability of Cu NPs

**Figure 6:**
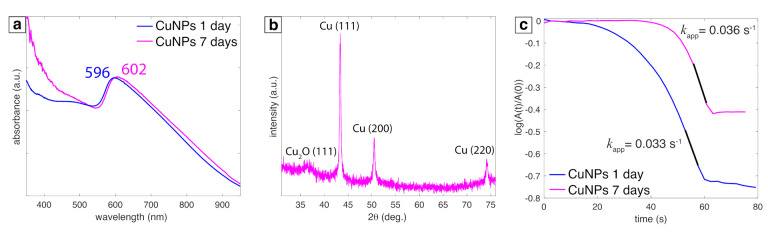
(**a**) UV-vis absorbance spectra measured at 1 day (blue) and 7 days (magenta). (**b**) XRD spectra for 7 day sample. (**c**) Time dependence of the absorbance of *p*-nitrophenolate ions at 400 nm for 1 day (blue) and 7 days (bright pink) samples; black portion of line is where $k_{\mathrm{app}}$ (s${}^{-1}$) is extracted.

Figure 6 compares the UV-vis absorbance, XRD spectrum, and PNP kinetics for 1 day (24 h) and 7 day samples. The Cu SPR peak observed for 7 day sample was only slightly red shifted compared to 1 day sample (Figure 6a), indicating no significant oxidation of Cu NPs. No changes were observed in the XRD peaks for 7 day samples (Figure 6b) compared 1 day samples (Figure 2), further confirming no or insignificant oxidation of Cu NPs. Accordingly, similar rate constants were obtained for 1 and 7 day samples for the PNP reduction reaction (Figure 6c). Collectively, these data indicate that the LRL Cu NPs are highly stable to oxidation.

## 4. Discussion

The production of Cu metal NPs using LRL requires both an organic solvent and air-free conditions, as no laser-induced conversion of Cu(acac)_2_ precursor was observed when the solvent was water or when the precursor in IPA/methanol mixture was irradiated under ambient conditions. This result underscores the importance of minimizing reactive oxygen species formation during LRL, which was also needed to effectively control the sizes of Au NPs [35] and enable formation of Ag-containing NPs [41,44,45]. The important role of reactive oxygen species has also been noted in PLAL synthesis of Cu NPs, where air removal completely eliminated formation of the highly oxidized CuO phase during PLAL in water [28]. Collectively, these results highlight similarities in the chemical reaction pathways induced during both PLAL and LRL that can lead to metal NP oxidation, as well as the potential for the same strategies to mitigate oxide formation.

The formation of substantial quantities of sub-10 nm Cu NPs in our LRL synthesis is similar to previous LRL results for Au and Ag NPs using well-controlled solvent chemistry [35,45], whereas the large Cu NPs up to ∼100 nm in diameter resemble the large Fe oxide NPs obtained from LRL of ferrocene in hexane [46]. The formation of two distinct size distributions of Cu NPs strongly suggests the participation of two different reaction mechanisms [33], although specific identification of these mechanisms is beyond the scope of this work. Nevertheless, on the basis of the PLAL literature demonstrating that substantial control over Cu NP size and morphology can be achieved by changing solvent mixtures [27,28,29,30], we anticipate that further exploration of different solvents will enable better control over Cu NP sizes using LRL. Finally, we note that the catalytic activity of the LRL Cu NPs to PNP reduction is comparable to that reported for PLAL Cu NPs [30], despite the presence of a thick carbon shell around our NPs. This result indicates that the carbon shell is sufficiently permeable to allow for catalytic reactions to take place and suggests that our LRL Cu NPs may have additional catalytic applications in areas, such as CO_2_ reduction or cross-coupling.

The formation of a substantial carbon shell around the LRL Cu NPs after termination of laser irradiation strongly suggests that the carbon formation is not entirely attributable to direct laser-induced solvent decomposition, as with PLAL in organic solvents [16,17,27,28]. Moreover, the lack of carbon shell formation during LRL of Fe(acac)_3_ in water [47] indicates that the acetylacetonate ligands from the precursor are not sufficient to induce carbon shell formation in LRL. On the basis of these results, we can speculate that the carbon shell is formed from catalytic activation on the Cu NP metal surfaces of long-lived solvent and ligand byproducts produced during laser irradiation. Cu metal is known to be a highly active catalyst in carbon-carbon cross-coupling reactions [19] and induces graphene growth from aliphatic alcohols under low-temperature CVD conditions [60]. Hence, we anticipate that the active bare Cu surfaces present immediately following laser irradiation catalyze the formation of the carbon shell. Although carbon shells are widely known to effectively protect transition metal NPs from oxidation [61,62,63], the evident permeability of the carbon around the LRL Cu NPs on the basis of their high catalytic activity suggests that a thin Cu_2_O layer around the Cu NPs also contributes to their observed stability [61].

## 5. Conclusions

We have reported the first synthesis of air-stable Cu NPs using the laser reduction in liquid (LRL) approach. Both small (<10 nm) and large (up to 100 nm) spherical NPs were observed, which primarily consisted of Cu${}^{0}$ metal on the basis of XRD and XPS analysis, although a Cu_2_O shell around the large NPs may be present. The Cu NPs exhibit remarkable stability over 7 days on the basis of the lack of significant changes observed in the UV-vis absorbance and XRD features and the similar rate constants obtained for PNP reduction. The LRL Cu NPs compare favorably with PLAL-synthesized Cu NPs in terms of Cu${}^{0}$ content, stability, and catalytic activity. The insights into LRL of Cu${}^{2+}$ gained in this manuscript can be extended to other metals for which oxidation of NPs is commonly observed during PLAL or LRL. Finally, further development of the synthesis conditions, such as conducting LRL in a flow setup, could increase Cu NPs yield to produce heterogeneous catalysts for applications, such as cross-coupling reactions and CO_2_ reduction.

## Data Availability

Data presented in this study are available on request from the corresponding author.
